# Enhancement of second-harmonic generation through Brillouin zone folding in a waveguide-coupled metasurface

**DOI:** 10.1515/nanoph-2025-0167

**Published:** 2025-09-03

**Authors:** Tsafrir Abir, Tal Ellenbogen

**Affiliations:** Raymond and Beverly Sackler Faculty of Exact Sciences, School of Physics and Astronomy, 208982 Tel-Aviv University, Tel-Aviv, 6779801, Israel; Center for Light-Matter Interaction, Tel-Aviv University, Tel-Aviv, 6779801, Israel; Department of Physical Electronics, School of Electrical Engineering, Tel-Aviv University, Tel Aviv, 6997801, Israel

**Keywords:** nonlinear, nonlocal, guided mode resonance, plasmonic, lattice resonances, dimerized metasurface

## Abstract

Metasurfaces have become significant platforms for optical manipulation, yet unlocking their full potential for nonlinear optics requires novel mechanisms to control and enable frequency conversion processes. This study demonstrates how structural dimerization in plasmonic metasurfaces coupled to waveguides can modify linear and nonlinear optical behavior via Brillouin zone folding. By introducing a centrosymmetric unit cell design featuring two mirrored split-ring resonators, we allow guided modes that were previously below the light line to appear as guided-mode resonances. These resonances facilitate nonlocal modes, which are present as distinct narrow transparency windows. Although centrosymmetric dimerized design typically forbids far-field radiation through quadratic nonlinear interactions, we observe notable second-harmonic generation – not merely through symmetry breaking at oblique incidence, which proves insufficient, but rather with the support of a nonlocal mode. The excitation of a collective mode at the pump frequency provides a strong nonlinear response by mediating the formation of a net dipole moment at the second-harmonic frequency, enabling far-field radiation that is otherwise forbidden. This synchronized action among split-ring resonators leads to observable second-harmonic generation, confirmed by both experimental evidence and simulations. Our results indicate that dimerized metasurfaces represent a versatile platform for harnessing collective modes in nonlinear interactions. This motivates further research and suggests promising applications in advanced nonlinear photonic devices.

## 1 Introduction

Optical metasurfaces, virtually flat arrays of nanostructures known as meta-atoms, have transformed our ability to manipulate light [1]. These ultra-thin interfaces achieve precise electromagnetic control through subwavelength abrupt interactions with the engineered surface, unlike traditional optical elements that usually control light by phase accumulation over long distances [2]. In recent years, they have also attracted attention for nonlinear optics [3], [4], where materials display nonlinear response at high optical intensities, allowing wave mixing and frequency conversion. Metasurfaces can manipulate and enhance such nonlinear processes through strong field confinement, engineered resonances, and control over the artificial nonlinear tensor [5], [6], [7].

In plasmonic metasurfaces, where the meta-atoms are metallic, nonlinear interactions originate from the asymmetry of the metal–dielectric interfaces [8]. The tightly confined localized surface plasmon resonances (LSPRs) significantly enhance light–matter interactions [9], and the efficiency of the nonlinear processes can be optimized by carefully designing the meta-atoms’ geometry and spatial arrangement. These degrees of freedom also offer precise control over phase, amplitude, and polarization to generate a desired nonlinear wavefront [10], [11], [12]. Meanwhile, the subwavelength thickness of the metasurface alleviates the need for a cumbersome phase-matching at the cost of interaction length. Consequently, significant research has focused on various strategies for further enhancement of the nonlinear response, including utilizing nonlocal, collective modes of the metasurface [13], [14], [15].

In plasmonic metasurfaces, the most studied nonlocal modes are surface lattice resonances (SLRs), which stem from Rayleigh–Wood anomalies (RAs) [16]. These narrow and dispersive resonances have been shown to significantly enhance nonlinear frequency conversion processes [17], [18], [19], [20], [21]. Guided mode resonances (GMRs) offer an alternative method for achieving collective nonlocal responses in metasurfaces [22], [23], [24]. These modes are formed by the diffractive coupling between free-space radiation and guided modes [25]. Recent experiments with gold split-ring resonators (SRRs) on TiO_2_ waveguides demonstrate how GMR-induced nonlocal modes enhance second-harmonic generation (SHG) [26]. These also significantly improved entangled photon pair generation through spontaneous parametric downconversion from a LiNbO_3_ thin film patterned with a metagrating [27].

GMR-based nonlocal modes offer several advantages over SLRs relying on RAs. GMRs do not require index-matching of the metasurface’s background medium [28], simplifying integration with other photonic structures. GMRs provide dual tunability through both the waveguiding structure and metasurface periodicity. Moreover, the metasurface design can control the diffractive coupling to guided modes. For example, asymmetric designs can introduce extrinsic chirality, enabling coupling to orthogonally polarized GMRs and enhancing difference-frequency generation [29]. In the following, we show how Brillouin zone folding in a dimerized metasurface enables a GMR that facilitates an otherwise forbidden second-harmonic generation. We demonstrate that this collective mode overcomes the limitations of both the centrosymmetric design and simple oblique incidence by transforming the symmetry of the fundamental excitation consequently modifying the SHG, allowing it to radiate efficiently to the far-field.

## 2 Design principles

Assuming the guided modes remain relatively undisturbed by the metasurface, the GMR condition closely follows the in-plane momentum-matching between the diffracted wave and the guided mode propagation vector

(1) $$\beta_{\text{T}{\text{E}}_{M}/\text{T}{\text{M}}_{M}}=k_{m_{1},m_{2}}^{\|}=k_{\text{inc}}^{\|}+m_{1}b_{1}+m_{2}b_{2},$$

where $\beta_{\text{T}{\text{E}}_{M}/\text{T}{\text{M}}_{M}}$ is the propagation vector of either the transverse electric (TE) or magnetic (TM) Mth-order guided modes, respectively. $k_{m_{1},m_{2}}$ and **k**_inc_ are the wavevectors of the diffraction order (*m*_1_, *m*_2_) and the incident field, respectively, with the ‖ superscript indicating the vectors’ components parallel to the metasurface’s plane. The reciprocal lattice vectors **b**_1_ and **b**_2_ determine the available diffraction orders, while unit cell composition governs the coupling efficiency.

**Figure 1:**
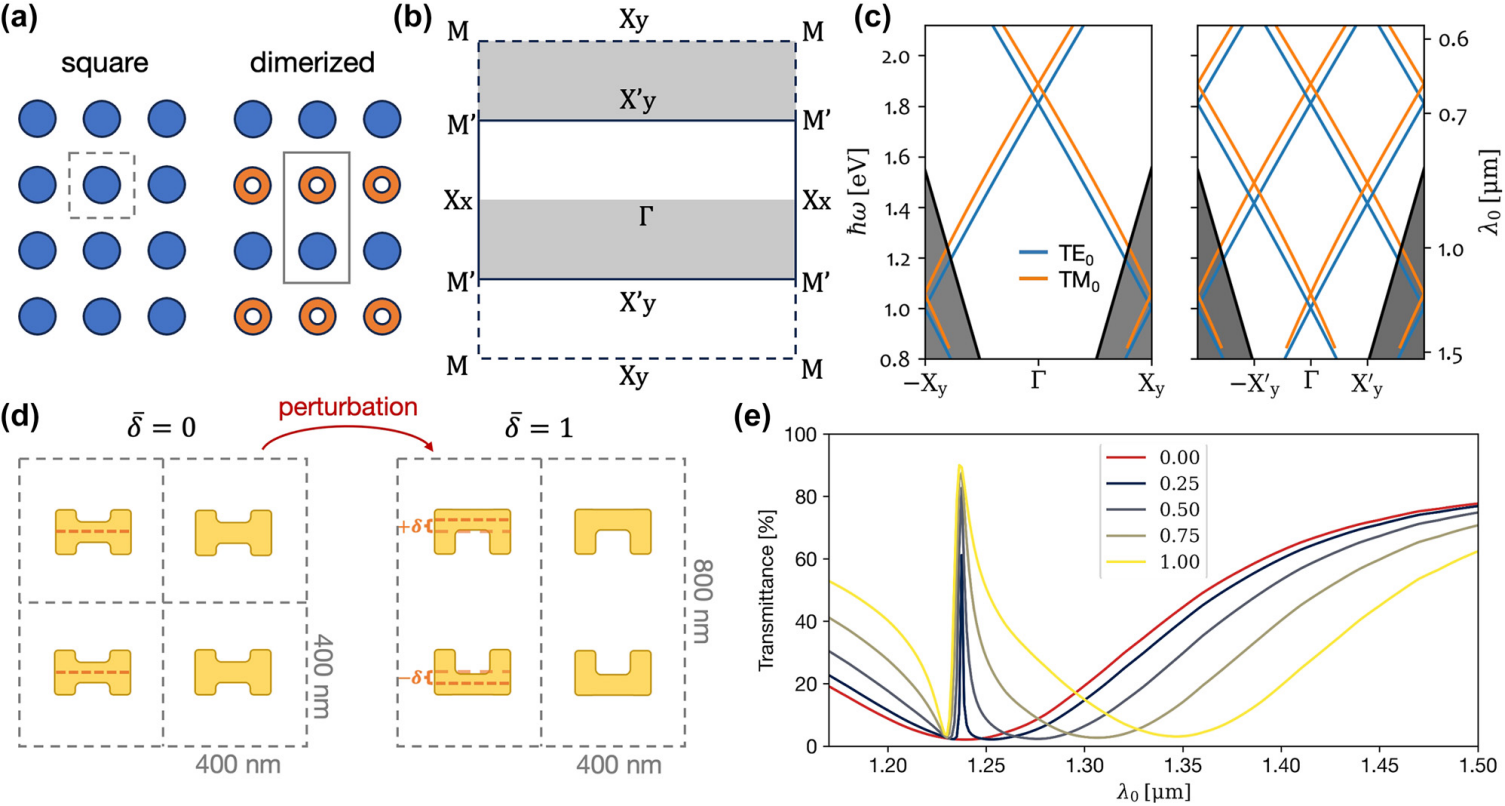
Brillouin zone folding through metasurface dimerization. (a) Schematic description of metasurface dimerization. (b) First Brillouin zone of the unperturbed and perturbed metasurfaces in (a). The dashed square and the solid rectangle define the reciprocal unit cells of the square and dimerized lattices. (c) Calculated mode dispersion for square and dimerized metasurface designs on a 255 nm thick SiN slab waveguide on glass. (d) Illustration of the structural perturbation to achieve a dimerized design by shifting the meta-atoms’ horizontal bar up and down in alternating rows. (e) The TE polarization transmittance spectra from simulations with different values $\bar{\delta }$ .

In dimerized metasurfaces containing two distinct elements per unit cell, as illustrated in Figure 1a, the increased periodicity folds the squared first Brillouin zone (FBZ) into a smaller rectangle in reciprocal space (Figure 1b) [30]. As a result, modes previously located in X_y_ high-symmetry point fold toward the Γ point. Figure 1c shows how guided modes, once below the light lines near the FBZ edges, are copied inwards, placing them above the light line to form GMRs. This concept of Brillouin zone folding parallels recent developments in quasi-bound states in the continuum (q-BICs) [31], [32]. A q-BIC leaks to free space only when perturbed; otherwise, it remains protected by symmetry. A key distinction is that guided modes do not exist in the continuum. Instead, they are brought to the continuum by translation symmetry breaking to form GMRs [14]. Nevertheless, these phenomena share characteristics and are analogous in many practical aspects.

Our study begins with numerical modeling of plasmonic metasurfaces on a 255 nm thick Si_3_N_4_ (SiN) waveguiding slab. We consider a square metasurface of “H”-shaped gold meta-atoms dimerized by applying a structural perturbation, as illustrated in Figure 1d. In alternating rows, the meta-atoms’ horizontal bar is shifted upward and downward by $\bar{\delta }=\pm \delta /32.5\;\text{nm}$ , such that the unit cell contains two meta-atoms, mirror images of one another. This gradual perturbation may increase until two inverted SRRs form in the unit cell ( $\bar{\delta }=1$ ). The transmittance spectra presented in Figure 1e reveal the formation of GMRs as transparency windows on the broader resonance of the localized mode. While increasing the perturbation, we can see how the newly formed GMR becomes broader, a characteristic of a q-BIC.

In the following, to obtain efficient nonlinear scattering [33], our experimental work focuses on the design with $\bar{\delta }=1$ , i.e., a dimerized metasurface of gold SRRs. It is important to note that while we chose a dimerization scheme that restores centrosymmetry, this is just one of many possibilities. Other periodic perturbations, such as displacing the SRRs, could be employed to fold the Brillouin zone while retaining the original noncentrosymmetric design. Furthermore, engineering the metasurface along the *x*-direction could enable coupling to other GMRs. However, we deliberately focus on this simpler, one-dimensional dimerization to clearly isolate and analyze the specific physical mechanism associated with folding the X_y_ point to the Γ point.

## 3 Experimental results

**Figure 2:**
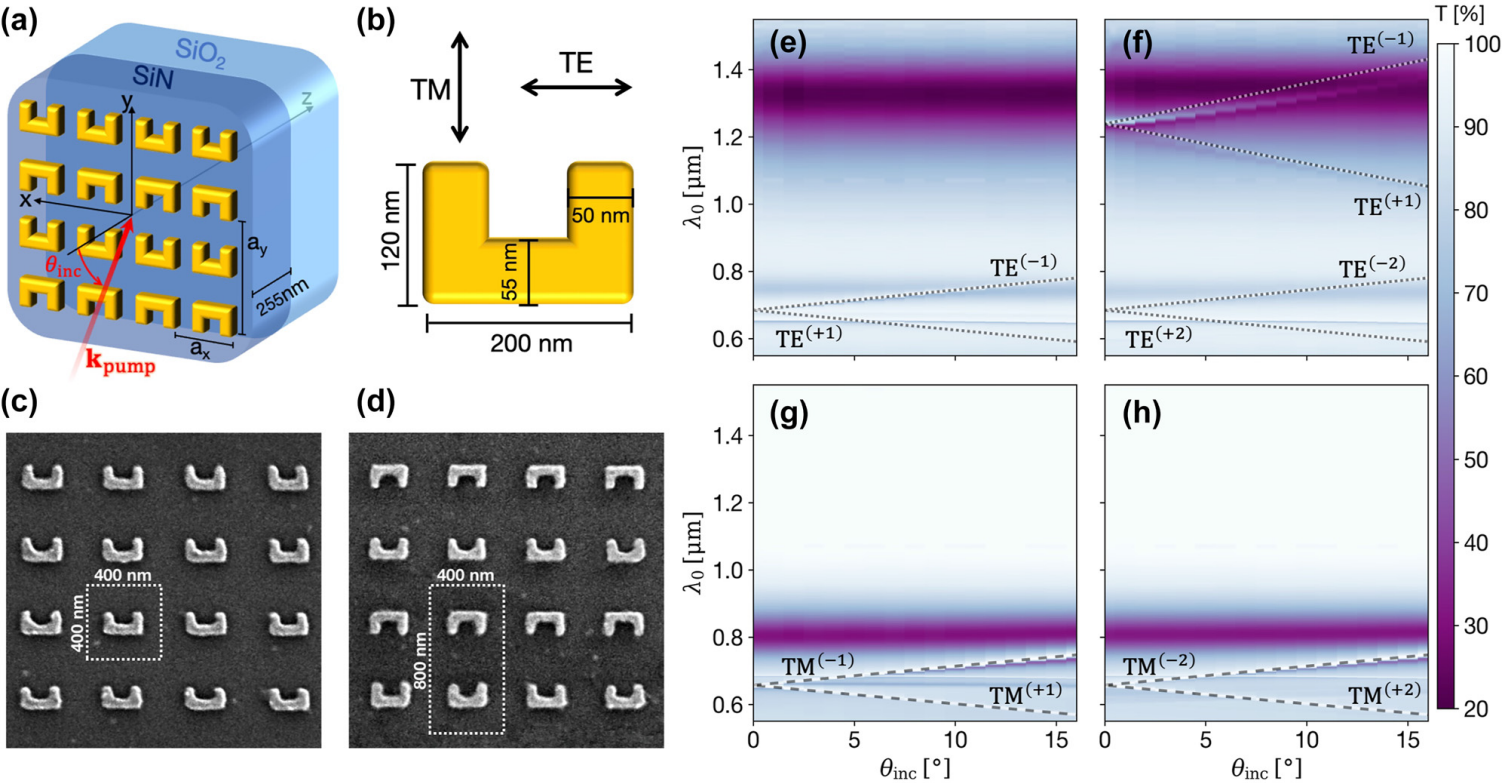
Experimental realization and linear characterization. (a) Schematic description of dimerized SRRs metasurface on a SiN waveguiding slab. (b) Illustration depicting the typical dimensions of the SRRs in fabricated metasurfaces and the definition of polarizations in the experiment. (c) and (d) show SEM images of resulting square and dimerized metasurfaces, respectively. (e,f) and (g,h) Angle-dependent transmittance spectra for TE and TM polarization, respectively, where the left panels present the results for the square metasurface and the right panels for the dimerized metasurface. The calculated GMRs’ dispersion lines are added and labeled for guidance.

We utilize a PECVD-grown SiN film that is 255 nm thick on a fused silica substrate on which a plasmonic metasurface is fabricated, as illustrated in Figure 2a. The waveguiding slab supports only the fundamental TE and TM-guided modes, as indicated in Figure 1c. For this reason, the subscript labeling the guided modes’ order will be omitted. Two metasurface designs measuring 100 µm by 100 µm were fabricated using e-beam lithography, followed by the evaporation of 2 nm of Ti for adhesion and 40 nm of gold (see Supplementary Material for methods’ details). All SRRs share similar dimensions, depicted in Figure 2b. Figure 2c and d present SEM images of the fabricated metasurfaces after liftoff and cleaning. The first is a reference metasurface design that has a square lattice with *a*_*x*_ = *a*_*y*_ = 400 nm. The second is the dimerized SRRs metasurface, obtained by periodically rotating the SRRs by 180° in every second row. This means that the periodicity in one dimension is doubled, i.e., *a*_*y*_ = 800 nm.

### 3.1 Linear characterization

Initially, we perform linear optical characterization by employing angle and polarization-resolved transmittance measurements (see Supplementary Material for setup detail), with the y-z plane as our plane of incidence. The resulting spectra are presented in Figure 2e–h, and the calculated GMRs’ dispersions are plotted for guidance. Away from the GMRs, the two metasurfaces present similar optical responses, resulting from the spectrally broad dispersionless local modes of the metasurfaces. The GMRs appear as finer dispersive spectral features. The most distinctive difference between the optical response of the two metasurfaces appears at TE polarization. The GMR resulting from the Brillouin zone folding in the dimerized metasurface resonates at longer wavelengths previously unreachable through the diffractive coupling. It is manifested as a narrow transparency window intersecting the broad fundamental LSPR. It deviates from the dispersion line as the spectral overlap with the localized mode increases. This deviation results from guided-mode dispersion that can no longer be considered unperturbed by the metasurface on resonance.

At the shorter wavelengths region of the spectra, the metasurface’s nonlocal modes are excited similarly. In addition to the angle-dependent GMRs, subtle horizontal features appear due to diffractive coupling in x to orthogonally propagating guided modes. It is worth mentioning that the unit cell has a broken mirror symmetry for the square metasurface that enables coupling to cross-polarized modes (coupling to both TE and TM modes regardless of the incident field’s polarization) [29]. Flipping the SRRs in the dimerized design restores this symmetry and turns off the cross-polarized coupling. It also leads to a centrosymmetric unit cell, impacting the metasurface’s ability to generate second-harmonic to the far-field, as detailed below.

### 3.2 Second-harmonic measurements

We now examine the quadratic nonlinearities in both designs. To effectively excite the metasurfaces, the pump beam must be TE polarized, aligning its polarization with the base of the SRRs, as suggested by the linear response (Figure 2e and f). Consequently, the second-harmonic field is cross-polarized, aligning its polarization along the arms corresponding to TM polarization based on our selected plane of incidence. A tunable femtosecond laser (Coherent’s Chameleon and Compact OPO system, ∼140 fs, 80 MHz repetition rate) pumps the fabricated metasurfaces (see Supplementary Material for more details). Scanning through both wavelengths and incidence angles, we can map the nonlinear response and reveal the effect of the nonlocal modes on the nonlinear processes. Our system captures the forward scattered SHG, and the quantum efficiency-corrected total photon count (*N*_SH_) is used to provide us with the conversion efficiency

(2) $$\eta_{\text{SH}}=\frac{\omega_{\text{SH}}\hbar N_{\text{SH}}}{{TP}_{\text{pump}}^{2}\left(\omega \right)},$$

where the exposure time *T* and the photons’ energy *ω*_SH_ *ℏN*_SH_ are used to evaluate the average SHG power, which is normalized by the square of the averaged pump’s power $P_{\text{pump}}\left(\omega \right)$ . While it doesn’t capture the full extent of the conversion efficiency because some of the SHG is backward scattered, absorbed, and remains confined to the waveguide, it still provides a measure to compare the nonlinear responses of the studied designs.

To ensure a fair comparison, all experimental parameters, including the incident laser pulse duration and illumination spot size, were kept consistent for the measurements of both metasurface designs. We also note that *η*_SH_ is strongly dependent on the resonance Q-factor [29] and its interplay with the pulse duration. While the specific design used here was not optimized for this parameter (as evident in Figure 1e), it represents a critical degree of freedom that could be engineered to further enhance the nonlinear conversion.

**Figure 3:**
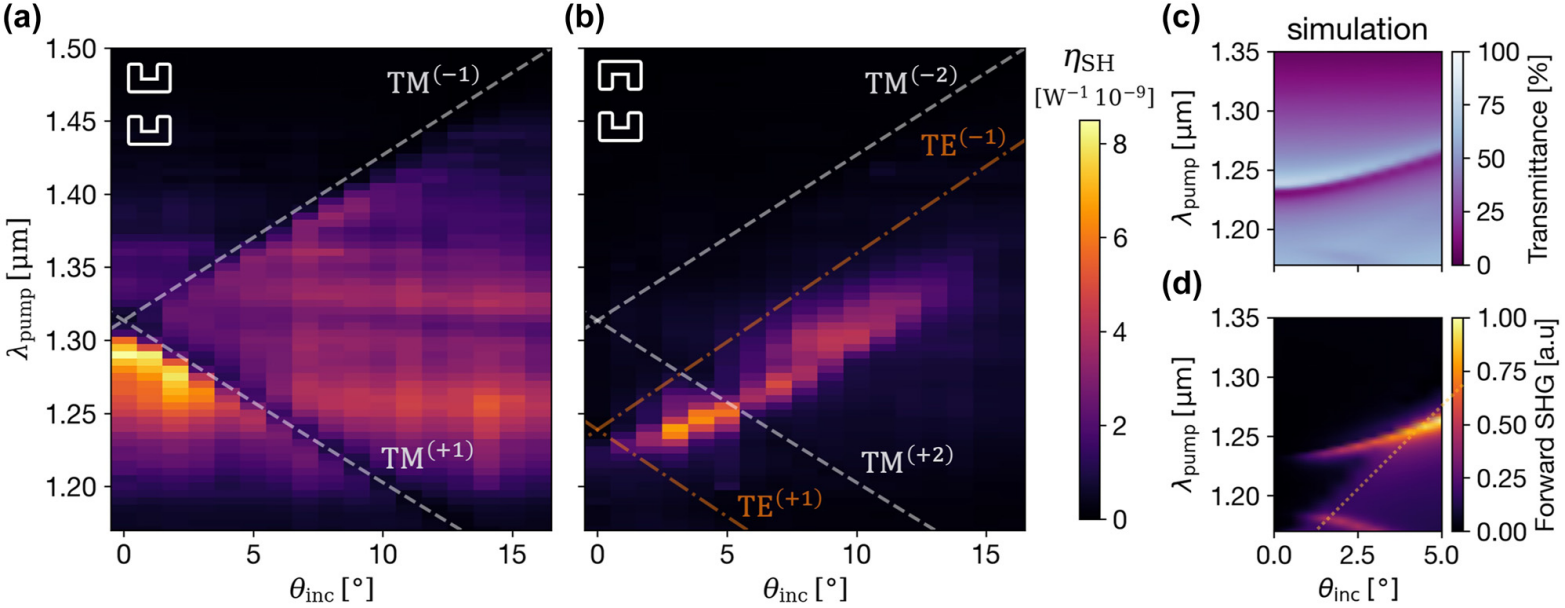
Angle and wavelength-dependent SHG conversion efficiency maps. (a) and (b) The results for square and dimerized SRRs metasurfaces, respectively, as indicated by the illustrations in the top-left corner. The TE GMR’s dispersion lines correspond to *λ*_pump_, and the TM GMRs are plotted for *λ*_SH_ = *λ*_pump_/2. Both share the same color scale. (c) and (d) The resulting transmittance and forward SHG (respectively) from simulations.

Figure 3a reveals the nonlinear response of the square metasurface. Due to the support of GMRs only at shorter wavelengths, the effects of the GMRs appear as dips running through the broader resonance of the dispersionless LSPR at the fundamental frequency. They indicate the coupling of the SHG to guided modes, as reported by previous studies [26], [29]. At smaller angles where GMRs intersect, peak enhancement of the conversion is observed due to the flattening of the band providing increased light–matter interactions.

In contrast, the measurement for the dimerized design, presented in Figure 3b, reveals strikingly different nonlinear response. As previously mentioned, the unit cell becomes centrosymmetric in the dimerized design. Alternatively, one can consider the two SRRs rotated by 180° generating second-harmonic with opposite phases that destructively interfere in the far-field [10]. This only occurs for the 0th-order nonlinear diffraction, not for the higher orders. For example, since *λ*_SH_ < *a*_*y*_ the 1st diffraction orders scatter second-harmonic to the far field. These are not captured by the numerical aperture of our experimental setup. Still, we experimentally measure a considerable SHG following the nonlocal mode at the fundamental frequency. We observe the same deviation from the plotted GMR dispersion as the transparency window in the linear response shown in Figure 2f. This suggests that the collective mode at the fundamental frequency synchronizes the meta-atoms to constructively scatter the second-harmonic to the far field. It is important to note that while the peak conversion efficiency is not higher than that of the noncentrosymmetric square lattice (Figure 3a), the result is significant because it demonstrates that the nonlocal mode can overcome the inherent symmetry constraints of the dimerized design.

## 4 Complementary simulations

To probe the contribution of the folded mode in the dimerized design, we model the photonic structure in COMSOL Multiphysics (refer to the Supplementary Material for further details). We used the Bloch boundary condition to define the unit cell; within it, we placed the two SRRs of the dimerized design on top of a SiN slab sandwiched between a semi-infinite SiO_2_ substrate and a free space. The simulation zone is terminated using PMLs. The nonlinearity is inserted into the simulation using the hydrodynamic model’s expression for the nonlinear surface currents on the SRRs’ facets [34]

(3) $$K_{2\omega }=\frac{i\omega }{n_{0}e}\left[\hat{t}\left(P_{\omega }^{⊥}P_{\omega }^{\|}\right)+\frac{\hat{n}}{2}\frac{3\omega +i\gamma }{2\omega +i\gamma }{\left(P_{\omega }^{⊥}\right)}^{2}\right],$$

where the induced polarization **P**_*ω*_ at the fundamental frequency *ω* is used with the metal’s free carriers density *n*_0_ and damping *γ*, the superscripts indicate the surface perpendicular and parallel vectorial components of the polarization, and $\hat{n}$ and $\hat{t}$ refer to resulting currents normal or transverse to the metallic–dielectric interfaces. The resulting surface current density is then used as a source in a simulation at 2*ω*.

We excite the modeled structure by TE polarized plane waves at a range of frequencies and angles where the distinctive nonlocal mode of the dimerized design appears. The resulting linear response for the pump wave, presented in Figure 3c, matches the experimental results in Figure 2f and validates the modeled system. The resulting normalized forward scattered SHG is presented in Figure 3d. In simulations, however, the higher-order diffraction orders also contribute to the calculated power, resulting in an increased SHG left to the substrate’s RA (yellow dotted line in Figure 3d). This increase is not evident in the experiments due to the limited numerical aperture of the measurement system. Overall, the main peak of the SHG in the simulation agrees with the *η*_SH_ obtained in the experiment.

### 4.1 Near-fields and currents

To connect the observed far-field enhancement with the underlying physics, we inspect the simulated near-fields and surface currents. Moreover, to provide a quantitative basis for this analysis, we perform a multipole decomposition of the induced currents. This decomposition confirms that the far-field radiation is dominated by the electric dipole moments. Specifically, the px component for the linear response and the py component for the SHG. The contributions from magnetic dipole and electric quadrupole moments are negligible (see Supplementary Information).

**Figure 4:**
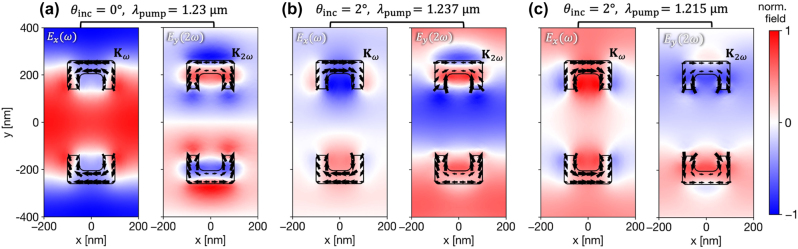
Near-fields and currents in the unit cell at the fundamental and second-harmonic frequency. The panels represent the simulation results for the parameters indicated above. (a) At normal incidence where the GMRs intersect. (b)/(c) Oblique incidence on/off resonance with the nonlocal mode, respectively. The results are all normalized and correspond to the fields mentioned in the inset labels.

To quantify the collective nature of the response, we calculate the electric dipole moment of each resonator in the dimer (**p**_1_ and **p**_2_) by integrating the simulated current density, $p_{i}\left(\omega \right)=-{\left(i\omega \right)}^{-1}\underset{V_{i}}{\int }J_{\omega }\left(r\right)d^{3}r$ . From these moments, we formulate the dimensionless “collectivity factor”

(4) $$\sigma =\frac{\left|p_{S}\right|-\left|p_{A}\right|}{\left|p_{1}\right|+\left|p_{2}\right|},$$

where the numerator represents the contrast between the symmetric (**p**_*S*_ = **p**_1_ + **p**_2_) and antisymmetric (**p**_*A*_ = **p**_1_ − **p**_2_) dipole combinations. The value of *σ* is bounded between +1 for a purely symmetric “bright” mode and −1 for a purely antisymmetric “dark” mode. We now use this metric to quantitatively interpret and compare the scenarios in Figure 4 (see Figure S6 for the complete evaluation of *σ*).

We sample at the normalized electric fields in the unit cell and the surface currents on the SRRs. These fields are taken from the plane 20 nm beneath the SRRs, and the current densities are sampled from the SRRs’ facets facing the waveguiding layer. For the TE-polarized fundamental field, *E*_*x*_ is used to map the fields, and for the TM-polarized SHG, we sampled *E*_*y*_. Figure 4 depicts three representative scenarios for comparison, with the relevant pump wavelength and incidence angle noted above. The panels qualitatively show how the symmetry of the fields and currents at ω influences the resulting nonlinear currents at 2ω.

At normal incidence (Figure 4a), the symmetric excitation field drives the linear currents contributing to $p_{x}\left(\omega \right)$ in unison, resulting in a perfectly symmetric fundamental mode with *σ* ^*ω*^ = 1. However, the second-harmonic response is governed by the geometry of the resonators. Since the two SRRs in the centrosymmetric unit cell are mirror images of each other, the symmetric fundamental field induces **K**_2*ω*_ along the resonator arms that are in opposite directions. This is clearly visualized in Figure 4a and results in two $p_{y}\left(2\omega \right)$ dipole moments that are equal in magnitude but opposite in phase. This physical opposition yields a perfectly antisymmetric collective mode (*σ* ^2*ω*^ = −1), confirming the destructive interference and complete cancellation of SHG in the far-field.

At oblique incidence (2°), when the pump is resonant with the nonlocal mode (Figure 4b), the physics changes dramatically. The GMR imposes a specific antiphase relationship on the fundamental currents to facilitate the diffractive coupling, leading to opposing $p_{x}\left(\omega \right)$ moments and a negative collectivity factor of *σ* ^*ω*^ ≈ − 0.35. This antisymmetric excitation, when processed by the effective quadratic nonlinearity of the inverted SRR pair, is transformed into a highly symmetric second-harmonic response. The resulting nonlinear surface currents on the resonator arms, which generate the $p_{y}\left(2\omega \right)$ moments, are driven in phase, as visualized in Figure 4b. This yields a “bright” collective mode with *σ* ^2*ω*^ ≈ 0.52, which radiates efficiently and produces the measured SHG.

Finally, for comparison, we consider the same 2° angle away from the GMR (Figure 4c). Without the resonant nonlocal interaction, the SRRs are primarily driven by the incident plane wave, which imposes a simple phase delay across the unit cell. This results in a partially symmetric linear response (*σ* ^*ω*^ ≈ 0.69). Following the same transformation as the normal incidence case, this symmetric portion of the excitation generates an antisymmetric second-harmonic response, visualized by the **K**_2*ω*_ on the arms of each respective SRR flowing in opposite directions. The resulting SHG mode is largely dark, with *σ* ^2*ω*^ ≈ − 0.32, leading to negligible far-field radiation. This clearly demonstrates that simple oblique incidence, without the resonant enhancement and phase-engineering of the GMR, is insufficient to generate a significant nonlinear signal.

## 5 Conclusions

In this work, we demonstrated how dimerization in plasmonic metasurfaces coupled to waveguides fundamentally alters linear and nonlinear optical responses through Brillouin zone folding. This folding enables guided modes previously below the light line to emerge as GMRs, providing an effective mediator for nonlocal metasurface responses. The emerging collective mode resonates at longer wavelengths, manifesting as a narrow transparency window. In the nonlinear regime, although our centrosymmetric dimerized design typically forbids quadratic interactions, we observe significant second-harmonic generation through a distinct mechanism. Symmetry breaking through oblique incidence alone proves insufficient for an observable nonlinear response. However, the excitation of the collective mode at the fundamental frequency substantially enhances the SHG by imposing a specific phase relationship between the resonators. This interaction results in a symmetric collective dipole moment at the second-harmonic frequency, which coordinates the far-field scattering. Computational simulations agree with the experimental results and provide insight into the near fields and currents, further validating the underlying mechanism. This work advances our understanding of nonlocal effects in waveguide-coupled metasurfaces, contributing to the ongoing development of nanoscale-integrated nonlinear devices.

While we explored a specific dimerization scheme that restores centrosymmetry, the principles demonstrated here are broadly applicable. The broad selection of design degrees of freedom offers numerous possibilities [35] for both linear and nonlinear interactions. Future work could extend to alternative designs and platforms, such as using inherently noncentrosymmetric dielectrics or nonlocal mode Q-factor optimization to further boost conversion efficiencies.

## Supplementary Material

Supplementary Material Details
