# Supplementary material for: Enhancement of second-harmonic generation through Brillouin zone folding in a waveguide-coupled metasurface
Source: Nanophotonics. 2025 Sep 3;14(18):3009–16. doi: 10.1515/nanoph-2025-0167 (PMC12442358; doi:10.1515/nanoph-2025-0167)
Supplement: Supplementary file 1 — Supplementary Material Details [file j_nanoph-2025-0167_suppl_001.pdf]

Supporting Information

Enhancement of Second Harmonic Generation through Brillouin Zone Folding in Waveguide Coupled Metasurface

Tsafrir Abir[1,2] and Tal Ellenbogen[2,3]

[1] Raymond and Beverly Sackler Faculty of Exact Sciences, School of Physics and Astronomy, Tel-Aviv University, Tel-Aviv 6779801, Israel
[2] Center for Light-Matter Interaction, Tel-Aviv University, Tel-Aviv 6779801, Israel
[3] Department of Physical Electronics, School of Electrical Engineering, Tel-Aviv University, Tel Aviv, 6997801, Israel

Sample Fabrication

The studied metasurfaces designs were fabricated on a PECVD grown Si_3N_4 waveguiding slab on fused silica substrate. It is 255 nm thick and its refractive index together with that of substrate [1] are plotted in Figure S1. These refractive indices are used in the evaluation of the guided mode's dispersion and in the finite element method simulations.

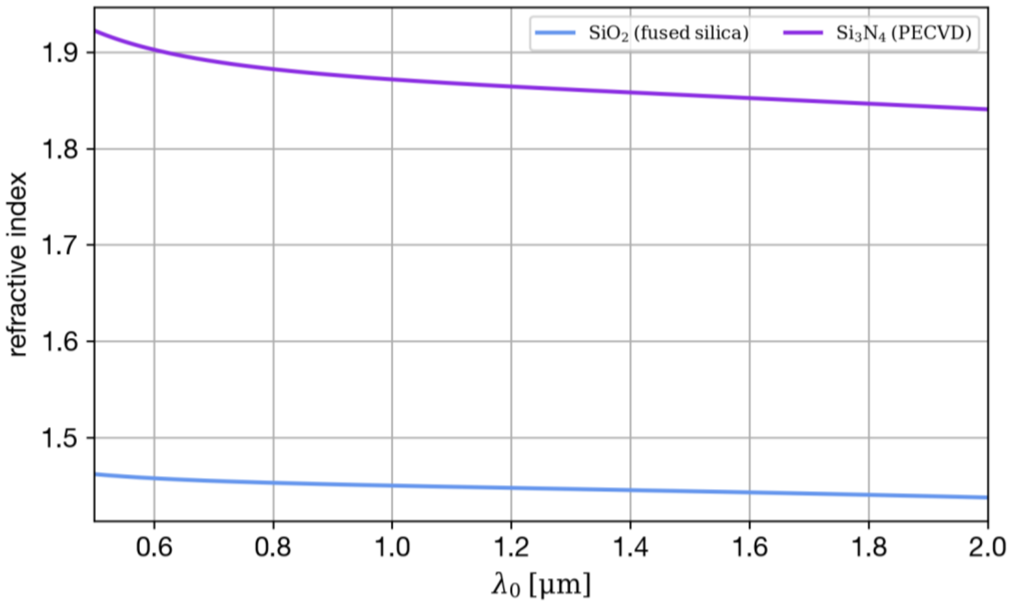

Figure S1 Refractive index of dielectrics used in the stratified waveguiding structure. The SiO_2 and Si3N4 labeled curves show the values for substate and waveguiding slab media, respectively.

E-beam lithography is used to pattern the waveguide with plasmonic metasurfaces. The different steps detailed below are schematically illustrated in Figure S2.

The substrates with the waveguiding films were cleaned right before each metasurface fabrication to remove any residues from the film deposition and dust accumulated while in storage. The samples were cleaned using acetone, IPA, and deionized water, followed by drying with an N_2 gun.

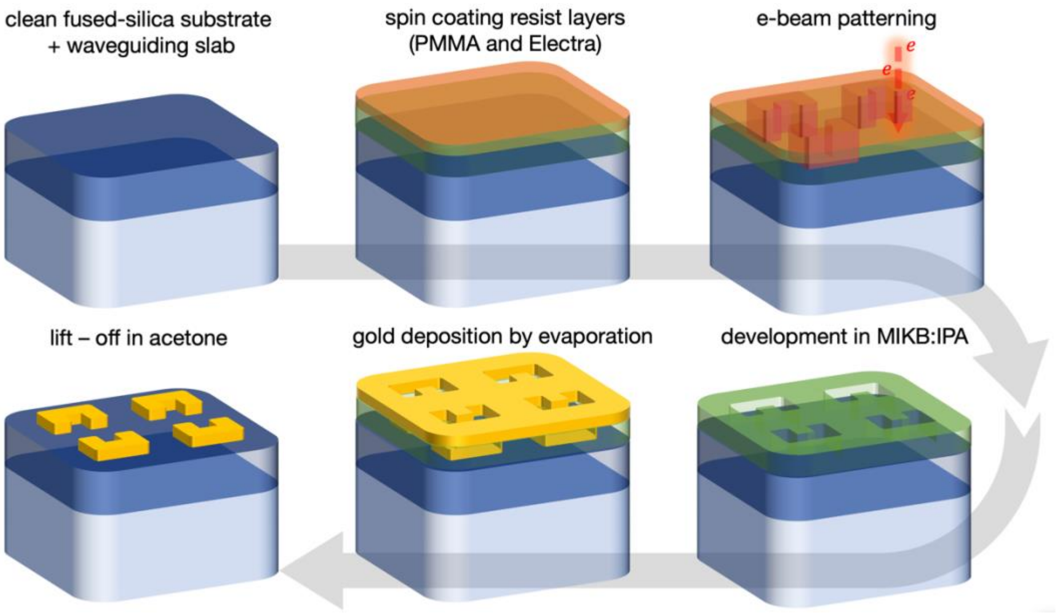

Figure S2 Graphical description of the steps in the plasmonic metasurface fabrication.

Resist Deposition

Initially, we applied a layer of positive resist (the beam exposure weakened the resist to eventually create a hole). We spin-coated MicroChem's 950 PMMA A2 solution (2% polymethyl methacrylate in anisole) at 2000 RPM (rounds per minute) for one minute, followed by soft/pre-baking on a hot plate at 180°C for one minute.

The insulating sample requires a conductive resist layer to ground the sample and avoid charge accumulation while exposed to the e-beam. We used Electra 92 (Allresist AR-PC 5090) spin-coated at 2000 RPM for one minute and then soft/pre-baking on a hot plate at 90°C for another two minutes.

E-beam Patterning

Using electron beam writer Raith 150 Two, the desired two-dimensional structure of the metasurface was patterned on the PMMA layer. We use 20 kV acceleration voltage and 10 μm size aperture to achieve the required resolution. The beam spot is about 20 nm in diameter, and the suitable resist exposure value was found to be 150 $\mu C/cm^2$.

After patterning, the Electra 92 layer is gently removed using deionized water. The Sample is then developed in MIBK:IPA (Methyl Isobutyl Ketone to IPA ratio 1:3) solution for one minute,

effectively dissolving the exposed PMMA regions. Then, the sample is washed with IPA and dried using an N2 gun.

Gold Deposition

The metal layers are then evaporated using an e-Beam Evaporator (VST TFDS-680) that uses a focused electron beam to heat and evaporate a target material. It offers precise control over the deposition rate and thickness, allowing for high-purity and uniform coatings in nanofabrication.

Initially, a 3 nm titanium (Ti) layer is evaporated, acting as an adhesion layer for the 40 nm evaporated gold (Au). We've found deposition rates of 0.5-0.8 Å/s provide a good compromise between accuracy and process duration.

Lift-off and Cleaning

The remaining resist is dissolved in acetone overnight, removing the metallic layers in the unpatterned regions. Additional removal is often required, accomplished by applying a gentle acetone flow to the sample. The final cleaning to prepare the sample for the experiment is done again with IPA, deionized water, and an N_2 gun.

Experimental Setups

Linear Characterization

To characterize the angle-dependent and polarization dependent linear response we employed the optical setup described in Figure S3. The sample was illuminated by a supercontinuum laser (NKT photonics Super-K compact), and transmittance was measured. The TM and TE polarization were interchanged using a polarizer (Orientation of the polarizations are marked in Figure S3). The sample was mounted on a rotating stage held to an XYZ stage. This provided us with the ability to perform measurements on a specific part of the sample for varying angles of incidence θ_{inc}. The light passing through the sample has been collected using Mitutoyo Apochromatic 20X Objective. Since the objective is infinity corrected, an additional tube lens (Thorlabs TTL200) with f=200 mm was used to create an image. The image is then duplicated using a beam-splitter, where one image is passed on to a CCD camera to monitor the part of the sample being measured and make the measurement process more convenient. The other image was directed onto an iris to limit the region from which the light is being measured. The light passing through the iris was focused on either of the two optical fibers. One fiber-fed Andor's Shamrock spectrograph with Newton EMCCD detector is used to measure the wavelength range of 600-950 nm. The second fiber was used for the range between 900-1500 nm with Ocean Optics NIR Quest. The spectral overlap between the two spectrometers ensures smooth stitching of the spectra. Switching the optical path between the two spectrometers was done by a flip mirror.

At each polarization and angle, a new reference measurement was taken by moving the sample so that only the part with the stratified structure (no metasurface) was measured. This has been done for both spectrometers. Performing these transmittance measurements provided us with the means to observe the effect of the metasurface and the diffractive coupling to the waveguide.

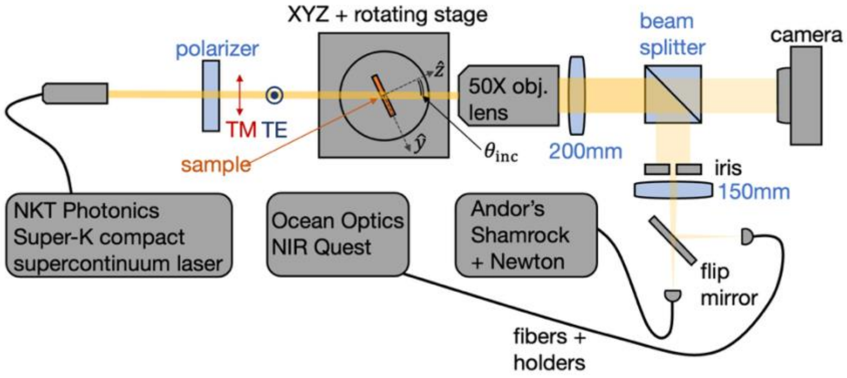

Figure S3 Schematic description of the optical setup used for the transmittance measurements. The details are specified in the figure and the in text.

Second-Harmonic Measurements

The optical setup used to measure the SHG is portrayed in Figure S4. The pump beam is sourced from Coherent's Chameleon and Compact OPO system. The system outputs ~140 fs pulses at a repetition rate of 80 MHz, with the OPO providing the means to tune the frequency of the pump. The beam was directed through a half-wave plate and a polarizer to control the polarization and power reaching the sample. The experiment was performed with the pump at TE polarization (as defined in Figure S3). The beam was focused by an achromatic doublet lens with a focal length of 200 mm (Thorlabs ACT508-200-C-ML). Before the sample, a 1100 nm longpass filter (Thorlabs FELH1100) was used to block any parasitic signals and a 10:90 beam splitter (Thorlabs - BS039) was placed to send part of the pump to an Ophir NOVA II power meter. Calibration measurements were made to correlate the power at the reflected and transmitted arms of the beam splitter for different wavelengths. The illuminating beam was weakly focused, and the power reaching the sample was kept in the range of 70-100 mW to avoid causing damage to the metasurface. The objective lens (MY20X-824 - 20X Mitutoyo Plan Apochromat Objective) collected the 0th-order transmitted pump beam and the generated SH, where 950 nm shortpass filter (Semrock FF01-950/SP-25) blocked the pump to allow only the SH to reach the sensitive scientific CMOS camera (Hamamatsu Orca Flash 4.0). A tube was used to reduce the amount of environmental scattered light that reaches the detector. The pixels in the spot's area of the nonlinear image were averaged and corrected by the camera's quantum efficiency and the square of the pump's power. This type of measurement was performed to measure the range of the pump frequencies and incident angles.

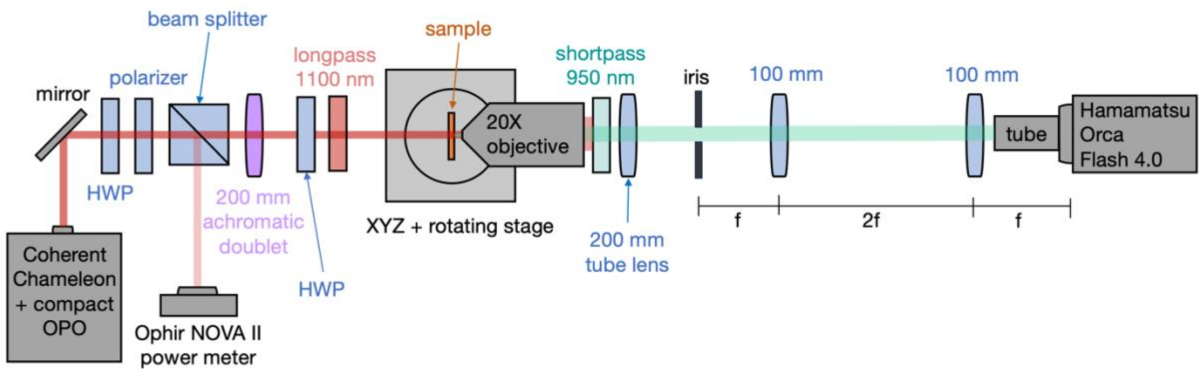

Figure S4 Schematic description of the optical setup used for the measuring the forward SHG. The equipment's' details are specified in the figure and the in text.

Numerical Modeling

Modeling Metasurface on a Waveguide

In our research, we employed Finite Element Method implemented in COMSOL Multiphysics, specifically its "Wave Optics Module."

Our numerical analysis assumes that the metasurface and waveguiding stratified structure are large enough to ignore edge effects. This allows the simulation of only a single unit cell (see simulation domain Figure S5) defined by the Bloch boundary condition, extending it infinitely in the lateral dimensions. The SRRs are modeled in the solver using the dimensions found for the fabricated metasurfaces together with gold's optical properties[2]. At the same time, the stratified structure is included by setting multiple planar interfaces and determining the optical properties of the volume confined between them using the refractive indices in Figure S1.

Perfectly matched layers (PMLs) above and below terminate the simulation region. This effectively sets the simulations with a semi-infinite substrate and superstrate. Nevertheless, setting the PMLs should be done carefully when simulating diffractive structures containing both radiating modes (diffraction orders) and evanescent modes (GMRs) or using steep incidence angles[3]. The systems we simulated include all of these, and thus, extensive convergence tests and fine-tuning of the PMLs parameters have been carried out to validate the solutions.

We used the periodic ports to approximate the system's excitation with plane wave illumination. By the definition of our coordinates system, the incident field propagates downward from the superstrate side, i.e., with negative values of k_z. These ports also evaluate the system's linear response by providing the absorptance, transmittance, and reflectance values.

While the solver's built-in module captures the linear response at given ω, it doesn't account for nonlinear interactions. To include the nonlinear frequency conversion process, the nonlinearity at the metallic interface needs to be modeled. We evaluated the nonlinear currents[4] excited on the

SRRs' facets using Eq.(3) from the main text. These currents are used as a radiation source for a following simulation at 2ω.

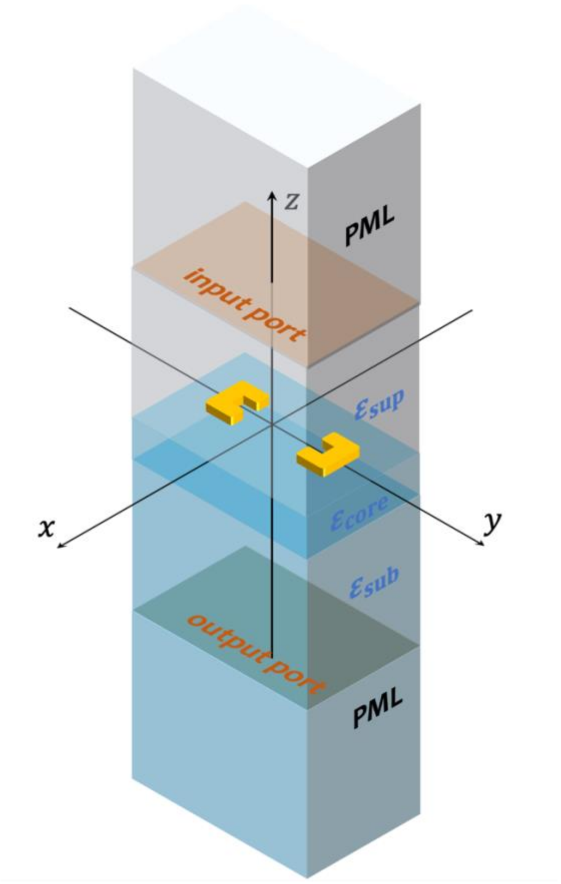

Figure S5 Illustration of the simulation layout.

Quantitative Multipole Analysis

Multipole Decomposition

The complex moments at a given frequency ω are calculated by integrating the simulated current density $\mathbf{J}_\omega(\mathbf{r})$ over the volume of each resonator (V_i). The leading moments under the long-wavelength approximation are [5]

$$\mathbf{p}_i(\omega) \approx -(i\omega)^{-1} \int_{V_i} \mathbf{J}_\omega(\mathbf{r})\, d^3r \qquad \text{Eq.S1}$$

$$\mathbf{m}_i(\omega) \approx \frac{1}{2} \int_{V_i} \mathbf{r} \times \mathbf{J}_\omega(\mathbf{r})\, d^3r \qquad \text{Eq.S2}$$

$$\mathrm{Q}_{i,\alpha\beta}(\omega) \approx -(i\omega)^{-1} \int_{V_i} \left[3\left(r_\beta J_{\omega,\alpha}(\mathbf{r}) + r_\alpha J_{\omega,\beta}(\mathbf{r})\right) - 2\mathbf{r} \cdot \mathbf{J}_\omega(\mathbf{r})\delta_{\alpha\beta}\right] d^3r \qquad \text{Eq.S3}$$

The complex current density $\mathbf{J}_\omega(\mathbf{r})$ is taken directly from the finite-element method solution and thus inherently includes all phase information arising from the incident field's wavevector (the Bloch phase) and the near-field coupling between resonators. It is also important to note that the magnetic dipole and all higher-order moments are origin-dependent. Therefore, all integrals were performed using a coordinate system centered on each respective resonator ($\mathbf{r}_{1,2} = [0, \pm 200\,\text{nm}\,, 0]$). To compare the relative contributions of these moments to the far-field radiation, their magnitudes must be converted to equivalent units ($[C \cdot m]$). The far-field amplitude of the magnetic dipole is proportional to $|\mathbf{m}|/c_0$, and that of the electric quadrupole is proportional to $k_0/\sqrt{15}|\mathrm{Q}|_{\mathrm{RMS}}$ where $k_0 = \omega/c_0$ and $|\mathrm{Q}|_{\mathrm{RMS}}$ is the root-mean-square magnitude of the tensor components.

Our analysis revealed that for the fundamental frequency the dominant moment is the electric dipole component p_x. The next largest contribution is the magnetic dipole m_z, whose equivalent dipole strength is 3-4 times smaller than p_x, corresponding to a power contribution of less than 12%. All electric quadrupole terms were found to be negligible. This justifies focusing the analysis at the fundamental frequency on the electric dipole moment p_x.

For the second-harmonic frequency (2ω**)** the analysis shows three significant moments of comparable magnitude: the electric dipole p_y, and the diagonal components of the electric quadrupole, Q_{xx} and Q_{zz}. However, the radiation pattern of a diagonal electric quadrupole tensor has a null for on-axis propagation ($\theta_{\mathrm{inc}} = 0°$) and scales with $\sin^2(\theta_{\mathrm{inc}})$. For the small angles of incidence used in our study, this angular dependence ensures their contribution to the 0th -order diffraction is negligible compared to the dipolar radiation. Therefore, the measured far-field SHG response is dominated by the electric dipole component p_y.This analysis confirms that examining the collective behavior of the electric dipole moments is sufficient for quantitatively describing the observed far-field nonlinear phenomena.

Collective Dipole Metrics

To quantitatively analyze the collective dipole response, we define two distinct but complementary metrics derived from the individual electric dipole moments of each resonator in the dimer ($\mathbf{p}_1$ and $\mathbf{p}_2$). The first is the Collective Dipole Strength ($|\mathbf{p}_S|^2$), this metric is the squared magnitude of the symmetric dipole combination, $\mathbf{p}_S = \mathbf{p}_1 + \mathbf{p}_2$. This value is directly proportional to the total power radiated into the far-field by the "bright" collective mode of the unit-cell. The second is the Collectivity Factor (σ), a dimensionless metric defined by Eq. (4) in the main. It characterizes the degree of phase synchronization and the symmetry of the collective oscillation.

Using both metrics is crucial for a complete physical picture. For example, a large $|\mathbf{p}_S|^2$ could result from one very strong resonator and one weak one (an unbalanced state with low σ), while σ with values approaching +1 indicates synchronized oscillations, even if the overall strength is weak. Analyzing both allows us to unambiguously identify a strong collective resonance.

Figure S6 map those metrics for the collective electric and magnetic dipole moments ($\mathbf{m}_S = c_0^{-1}(\mathbf{m}_1 + \mathbf{m}_2)$) at the fundamental frequencies and for the electric dipole at the second harmonic frequencies. For the excitation at ω we can recognize that despite the positive values of collectivity

factor for the magnetic moment along in Figure S6(b), its strength is order of magnitude smaller than the electric dipole. This further justifies focusing the analysis on the electric dipole moment, as previously noted.

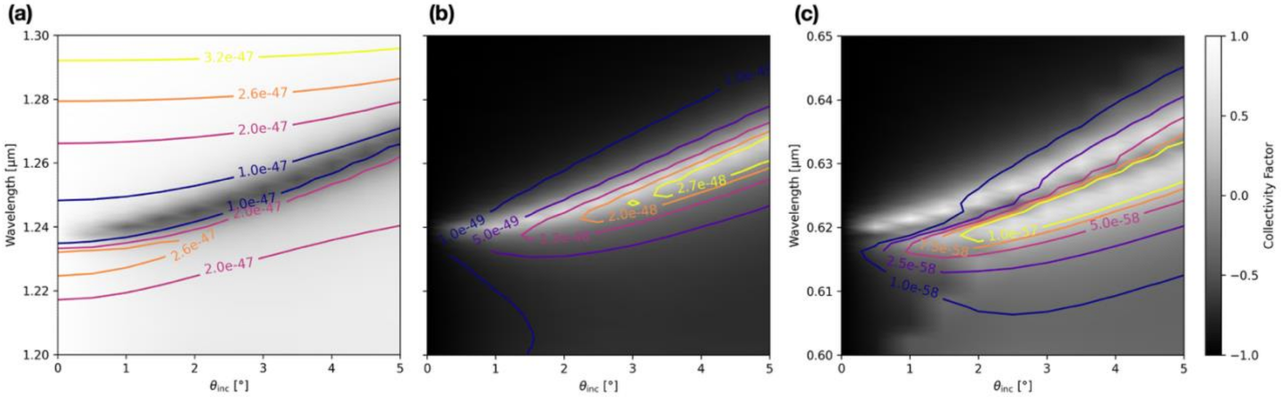

Figure S6 Collective dipole metrics of the leading dipole contributions for different incidence angles and wavelengths. The gray-scale map the dimensionless values of σ and the contour lines show the levels of the Collective Dipole Strength in units of $C^2 \cdot m^2$. (a) present the results for electric dipole at ω, (b) the results for the magnetic dipole at ω, and (c) the electric dipole at 2ω.

Figure S7 show a cross-section of Figure S6(a,c) at $\theta_{inc} = 0$ and 2, with circles indicating the relevant values for scenarios presented in Figure 4 and mentioned in section 4.1 in the main text.

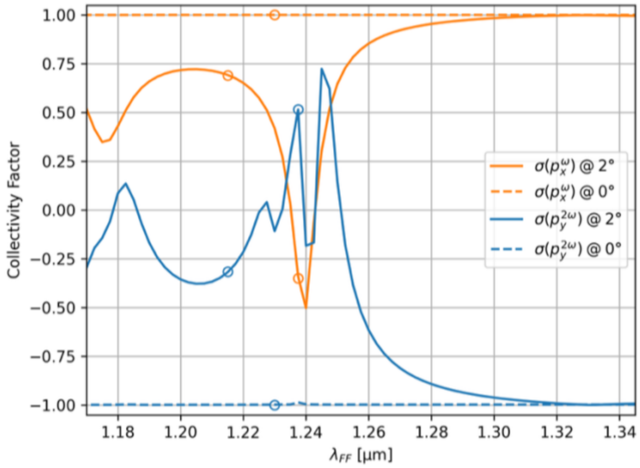

Figure S7 Collectivity Factor for the fundamental and second harmonic wavelengths. The circles mark the values for scenarios discussed in the main text.

References

[1] I. H. Malitson, "Interspecimen comparison of the refractive index of fused silica," *Josa*, vol. 55, no. 10, pp. 1205–1209, 1965.

[2] D. I. Yakubovsky, A. V. Arsenin, Y. V. Stebunov, D. Yu. Fedyanin, and V. S. Volkov, "Optical constants and structural properties of thin gold films," *Opt. Express*, vol. 25, no. 21, p. 25574, 2017.

[3] S. Erlandsson, "Evaluation, adaption and implementations of perfectly matched layers in COMSOL multiphysics," M. Thesis, KTH Royal Institute of Technology, 2020.

[4] C. Ciracì, E. Poutrina, M. Scalora, and D. R. Smith, "Origin of second-harmonic generation enhancement in optical split-ring resonators," *Phys. Rev. B*, vol. 85, no. 20, p. 201403, 2012.

[5] R. Alaee, C. Rockstuhl, and I. Fernandez-Corbaton, "An electromagnetic multipole expansion beyond the long-wavelength approximation," *Optics Communications*, vol. 407, pp. 17–21, 2018.
